# Generation of Human Bioartificial Tissues Using Agarose-Derived Biomaterials

**DOI:** 10.3390/ma19173645

**Published:** 2026-08-27

**Authors:** Fernando Campos, Jesús Chato-Astrain, Miguel Ángel Martín-Piedra, Óscar Darío García-García, David Sánchez-Porras, Miguel Etayo-Escanilla, Paula Ávila-Fernández, Ingrid Garzón, Miguel Alaminos

**Affiliations:** 1 Tissue Engineering Group, Department of Histology, School of Medicine, University of Granada, E18016 Granada, Spain; fcampos@ugr.es (F.C.); jchato@ugr.es (J.C.-A.); mmartin@ugr.es (M.Á.M.-P.); ogarcia@ugr.es (Ó.D.G.-G.); davidsp@ugr.es (D.S.-P.); metayo@ugr.es (M.E.-E.); pavila@ugr.es (P.Á.-F.); igarzon@ugr.es (I.G.); 2 Instituto de Investigación Biosanitaria ibs.GRANADA, E18012 Granada, Spain

**Keywords:** agarose, biomaterials, tissue engineering

## Abstract

**Highlights:**

- Agarose sources, extraction steps, and structural variants define its biomaterial performance.
- Physical, optical, and biomechanical properties of agarose hydrogels guide TE applications.
- Agarose-based scaffolds support biofabrication of diverse tissues, with several reaching clinical use.

**Abstract:**

Agarose is a thermoreversible, highly biocompatible polysaccharide increasingly used in tissue engineering (TE). Its molecular architecture, optical clarity, tunable mechanics, and chemical inertness make agarose hydrogels attractive scaffolds for generating bioartificial tissues by TE. This review summarizes current knowledge on agarose extraction, purification, structural variants, and physicochemical properties regarding gelation behavior, stiffness, porosity, and bioactivity. We discuss how agarose type and concentration critically determine hydrogel biomechanical and optical performance, influencing cell behavior and in vivo suitability. Although biologically inert, agarose can be functionalized or combined with fibrin, collagen, chitosan, and other biomaterials to enhance cell adhesion, proliferation, and differentiation. Diverse biofabrication approaches—including micromolding, bead production, 3D bioprinting, and de novo assembly of cells, biomaterials and bioactive factors—have enabled the generation of microtissues, organoids, and complex multilayered constructs. Agarose-based biomaterials allowed for the successful generation of bioartificial substitutes of cartilage, bone, adipose tissue, skin, cornea, oral mucosa, and the peripheral nerve, with several fibrin-agarose advanced therapy medicinal products (ATMP) already reaching clinical application, including the skin substitute UGRSKIN, the artificial cornea NANOULCOR and the palate mucosa BIOCLEFT. Together, current evidence positions agarose as a versatile and translationally relevant biomaterial for next-generation TE, warranting further exploration of its potential in additional therapeutic contexts.

## 1. Introduction

Agarose is a linear polysaccharide extracted from certain species of *Rhodophyceae* (red algae), particularly, the genera *Gelidium* and *Gracilaria* [1] and, to a lesser extent, *Ceramium*, *Acantkopeltis*, and *Pterocladia* [2]. In these algae, agarose and related polysaccharides form agar, which is a major constituent of the cell wall matrix providing mechanical stability, flexibility and resistance to the biomechanical forces found in their natural medium [3].

Agarose is currently employed in a wide range of applications with high economic and technological impact [4]. In the food industry, agarose is widely used as a thickener, stabilizer, and clarifying agent, as well as to improve the appearance and texture of desserts and bakery products [1]. In biomedicine, agarose is widely used as a component of bacteriological culture media, and as a matrix for research and diagnostic applications, especially for DNA separation by electrophoresis [5].

One of the most promising applications of agarose is the generation of bioartificial tissues and organs by Tissue Engineering (TE), and the use of agarose in TE is expected to increase significantly in the coming years. TE is an interdisciplinary field that applies the principles of biology and engineering to the development of functional substitutes for damaged tissue [6]. Its primary objective is to create bioartificial tissues or tissue substitutes capable of restoring, maintaining or improving tissue function. In most cases, bioartificial tissues generated by TE are composed of three key elements: cells, biomaterials and bioactive factors. These components are combined to generate a biological substitute that should biomimetically reproduce the structure and function of native tissues and organs, thereby promoting tissue regeneration [7]. In this regard, biomaterials used in TE should be highly biocompatible, and support cell attachment, proliferation and differentiation.

Due to their physicochemical properties, agarose hydrogels typically show good transparency, consistent gel strength and biocompatibility [8], and its use in TE has been demonstrated to favor cell proliferation and cell physiology [9]. Despite its excellent physicochemical properties, agarose is considered to be a bio-inert biomaterial that does not present any cell adhesion motifs required for cell–matrix interactions [10]. Since most cells attach to the surrounding extracellular matrix (ECM) via integrins and other adhesion proteins that are not present in pure agarose hydrogels, cells cultured within these biomaterials may require an adaptation period during which cells would actively synthesize pericellular ECM, allowing cells to actively interact with the biomaterial and respond to environmental stimuli [11]. Furthermore, a combination of agarose with other types of biocompatible biomaterials, such as fibrin [12], collagen [13] and chitosan [14], provides agarose biomaterials with ECM biomolecules, resulting in improved cell homing and proliferation [15].

On the other hand, different types of agaroses with different physicochemical properties and potential biomedical usefulness have been described to date [16], but the potential applications of each agarose type in TE still need to be determined.

## 2. Agarose Extraction and Purification from Natural Sources

**Figure 1:**
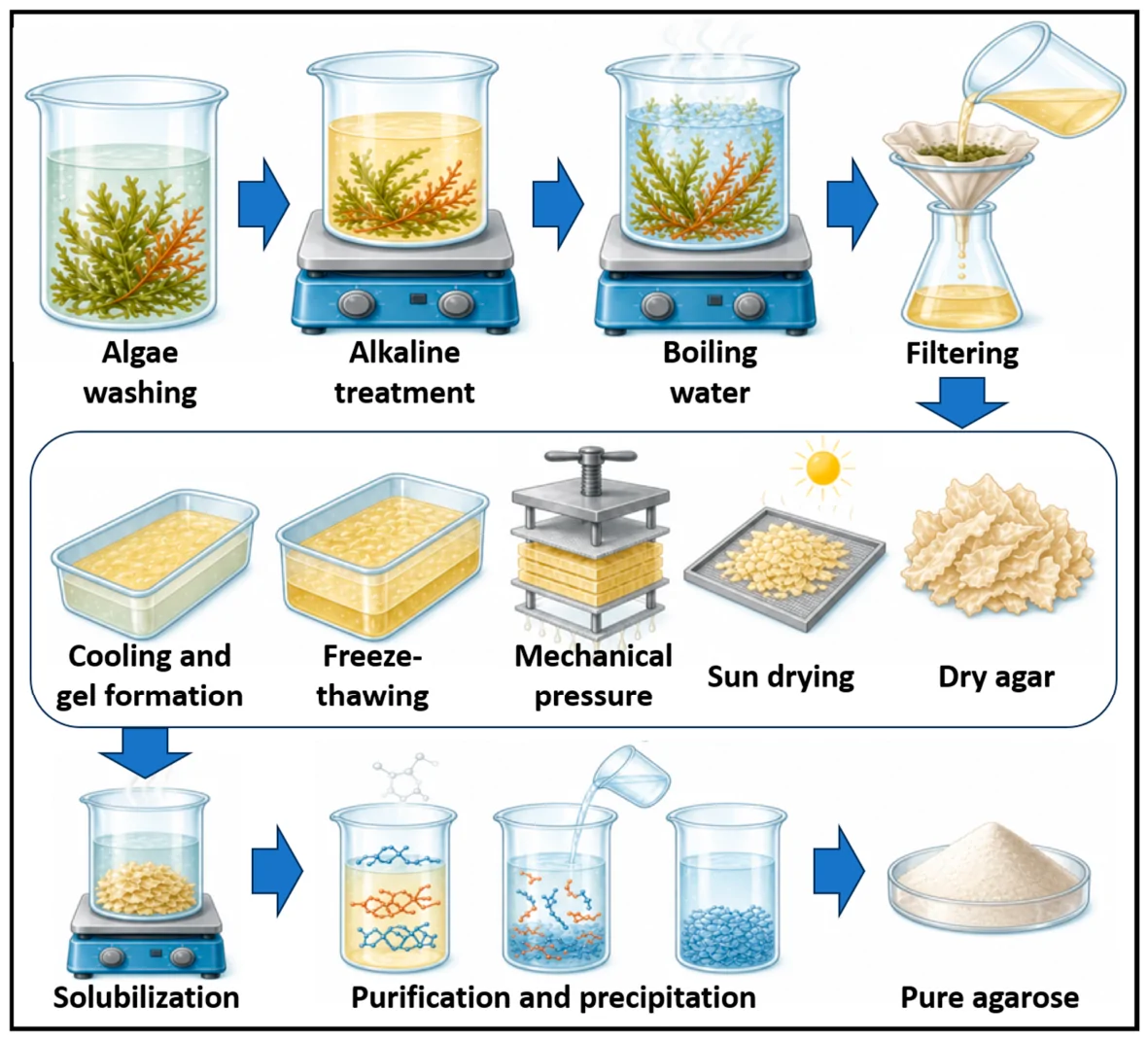
Steps typically followed for agarose extraction and purification from marine algae. Image generated using Illustrae Professional Scientific Illustration Platform at https://illustrae.co/ (accessed on 15 July 2026), and subsequently modified by the authors. This tool generates and iteratively refines scientific illustrations from user prompts, sketches, or images, which are then assembled and manually edited into the final figure.

Agarose is generally extracted (Figure 1) from diverse marine algae that contain high amounts of agar, which is a mixture of agarose and agaropectin, in their cell walls [4]. Once the different algae have been collected and cleaned, the first step of the agar extraction procedure is alkaline pretreatment, usually performed with sodium hydroxide. The objective of this procedure is to reduce the sulfate groups of the agar by inducing desulfation reactions that favor the gelling properties of agar. It has been demonstrated that the concentration and temperature used during this alkaline treatment can significantly influence agar yield and properties [17].

Following alkaline treatment, seaweeds must be submerged for several hours in boiling water to dissolve agar into the aqueous phase to generate an eluent that is enriched in solved agar, with approximately 70% of this agar corresponding to agarose [18,19]. Although other solvents different to water can also be used, water extraction is the most commonly used method for agar extraction and purification [4]. The resulting extract is then filtered to remove insoluble components and cooled to form agar gels [20]. Then, water is removed from the agarose gels using freezing–thawing cycles, mechanical pressure or other dewatering techniques, after which, the material is dried using hot air or sun exposure to obtain the agar product.

To generate pure agarose from agar, the final product must be further purified by removing agaropectin and other charged molecules using several purification procedures able to yield agarose with low sulfate contents and high gelling potential [1]. Due to the different solubility showed by agarose and agaropectin, precipitation methods using polyethylene glycol or other solvents are frequently applied to generate pure agarose materials [21], although other methods such as chromatography can also be used [22].

Although very little information is available on the effects of the different sources of algae on the chemical composition and properties of the final product, it is expected that the use of different species of algae or different varieties and geographical distributions of the same species can significantly influence the obtained agarose.

## 3. Agarose Modifications

Once extracted and purified, agarose can be modified by applying several enzymatic, physical and chemical modification methods able to tailor its properties [1]. By using enzymatic methods, agarose can be modified with desulfatases to induce desulfation, or can be oxidized for application in food packaging films [23]. By applying physical methods, agarose biomaterials can be subjected to micronization, homogenization, moist heat treatment or pulsed electric fields, among other methods, resulting in a product with specific properties and characteristics [1].

In turn, chemical modifications are widely used due to their reproducibility, simplicity and efficiency, enabling the introduction of functional groups into the agarose structure, resulting in a product with improved properties that can be optimized for diverse biotechnological and biomedical applications, such as melting behavior, gel strength, transparency, and emulsifying capacity. One of the most important applications of chemical modification is the generation of low-melting point agarose, which is frequently prepared by oxy-alkylation of agarose [24] or by other chemical treatments able to interfere with the formation of intermolecular hydrogen bonds during gelation. These modifications frequently involve the incorporation of hydroxyethyl or other chemical groups into the agarose chain, reducing intermolecular interactions, thereby lowering the gelling and melting temperatures of agarose biomaterials.

In addition, the use of octenyl-succinic anhydride has been reported to generate novel agar types with increased transparency [25]. Furthermore, methacrylate groups can be incorporated into the agarose structure using methacrylic anhydride to generate methacrylated agarose, a photo-crosslinkable agarose derivative able to generate covalent bonds using ultraviolet light exposure, thus resulting in a highly stiff biomaterial [26]. Furthermore, agarose can be functionalized with bioactive molecules, growth factors, RGD peptides, nanoparticles, lipids, or antimicrobial agents, providing this biomaterial with definite biological properties. Finally, agarose can be combined with other biomaterials to generate composite hydrogels able to exert the properties of both types of biomaterials. As agarose is considered to be non-adhesive for living cells, functionalization with bioactive molecules and the generation of composite biomaterials could contribute to improving cell attachment and development.

The possibility to tailor the properties of agarose through diverse biological, chemical and physical modifications to generate specific agaroses with definite properties makes agarose a highly promising biomaterial for use in biomedicine and, specifically, in TE.

## 4. Chemical Structure of Agarose

**Figure 2:**
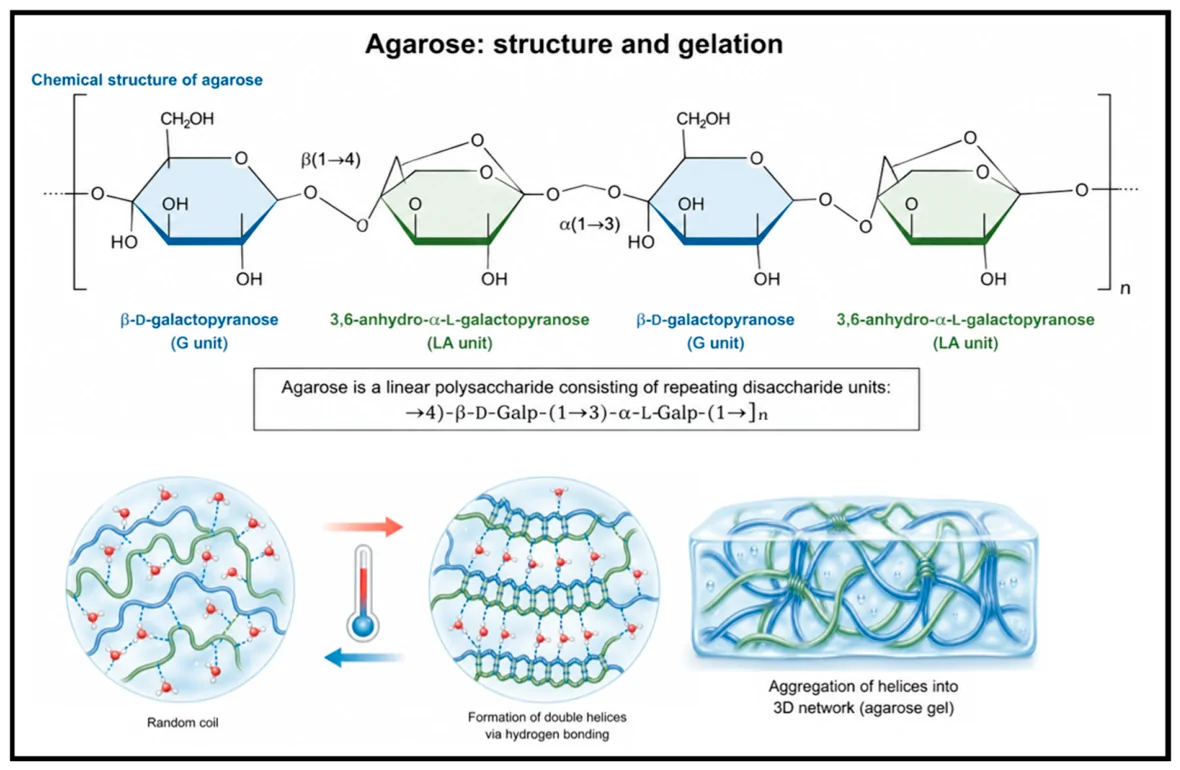
Chemical structure of agarose and schematic illustration of its thermoreversible gelation mechanism. Image generated using Illustrae Professional Scientific Illustration Platform at https://illustrae.co/ (accessed on 15 July 2026), and subsequently modified by the authors. This tool generates and iteratively refines scientific illustrations from user prompts, sketches, or images, which are then assembled and manually edited into the final figure.

Structurally, agarose consists of long linear chains of repeated agarobiose units, each composed by a combination of β-D-galactose and 3,6-anhydro-α-L-galactose residues linked by alternating β-(1→4) and α-(1→3) glycosidic bonds [8,27] (Figure 2). In normal conditions, a small percentage of these residues can be methylated or show sulfate and pyruvate modifications [15].

The agarose structure contains numerous hydroxyl groups that tend to form hydrogen bonds within the polymer network and with surrounding water molecules, resulting in a highly hydrophilic substance. At high temperatures, these hydrogen bonds are progressively broken, resulting in dispersion of agarose polymer chains, whereas low temperatures promote re-establishment of intermolecular hydrogen bonds, especially at specific points known as junction zones [5], leading to the formation of tightly arranged double helix structures able to form stable gels [1]. This particular property of agarose is known as thermoreversibility, while the difference between the gelation temperature and the melting temperature is referred to as thermal hysteresis [28].

The chemical architecture of agarose, especially the abundance of hydroxyl groups and the alternating β-(1→4) and α-(1→3) bonds directly governs its physical behavior. These structural features are the main ones responsible for controlling the capability of agarose hydrogels to form hydrogen-bonded junction zones, undergo thermoreversible gelation, and form double-helix aggregates that define the stiffness, thermal hysteresis, and gel strength of agarose hydrogels [29]. Thus, the physical properties discussed in Section 5 originate from the molecular interactions described here.

Although agaroses have not been properly classified, reports suggest the existence of several types of agaroses. All agarose types share the main features and functional characteristics of this biomaterial. However, significant differences exist among agaroses, which can substantially influence their behavior in biomaterial applications. In this milieu, physicochemical modifications of agarose, such as methylation, sulfation, or association with different molecules and factors, can significantly alter hydrogen-bonding patterns and helix formation, thereby modulating gelation temperature, thermal hysteresis, and overall gel strength [28]. Apart from the intrinsic differences derived from specific algae sources, these chemical variations can give rise to the different agarose types described below, each exhibiting distinct gelation behaviors and functional performance.

- Standard agarose. Most agaroses applied to routine applications can be considered as standard agaroses. In most cases, these agaroses have a gelation temperature of approximately 35–40 °C and a melting temperature of approximately 85–95 °C, and have low concentrations of sulphates.
- Low-melting point agarose [24]. This agarose is able to melt at approximately 60–70 °C and jellifies below 35 °C (usually, between 25 and 30 °C). These temperatures make this product more suitable for use in TE than standard agarose, as cells can be incorporated into the liquid product at physiological temperature, without altering cell viability. As explained above, this agarose is commonly synthesized by chemically modifying standard agarose to interfere with the formation of intermolecular hydrogen bonds during gelation.
- High-resolution agarose. This type of agarose has a small, uniform pore structure and can be efficiently used to separate specific molecules, especially DNA, by electrophoresis, with improved resolving potential. In most cases, this agarose is chemically identical to standard agarose, but specific agarose fractions with definite molecular weight can be selected, although it can also be obtained by partial chemical modification or purification of standard agarose using chromatography [1].
- High-strength agarose. Agarose biomaterials can be tuned and optimized to obtain a formulation with increased resistance, gel strength and biomechanical properties, as compared to standard agarose. To obtain this type of agarose, researchers can select and purify specific agarose fractions, reducing impurities or applying specific chemical modifications resulting in robust gels that are particularly suitable for applications requiring stiff agarose biomaterials.

## 5. Physical Properties of Agarose Hydrogels

In most cases, agarose biomaterials are obtained by researchers as a dry powder that is resuspended in a cold buffer, such as TAE (tris-acetate-EDTA), TBE (tris-borate-EDTA) or PBS (phosphate-buffered saline), and boiled to obtain a homogeneous solution that will jellify and solidify after cooling at room temperature [30]. A major advantage of agarose solutions is their compatibility with high-temperature autoclave sterilization, making them suitable for products intended for clinical use. The specific physical properties of these hydrogels will depend on several factors, with the agarose type and concentration being the most relevant [16], as these factors will significantly influence the porous structure of agarose hydrogels [5,16].

It has been described that the pore size of 1% agarose hydrogels is approximately 100 nm, which makes agarose an ideal platform for electrophoresis applications [31], although larger pores may be needed for TE purposes [32]. The porous structure of hydrogels is very important to allow diffusion of nutrients and oxygen, and for growth factors, cell migration and matrix deposition. In general, high-resolution agaroses produce finer meshes, suitable for small-molecule diffusion, while low-melting point agaroses tend to form wider and more open networks. Moreover, agarose hydrogels generally contain more than 99% water, enabling efficient diffusion of small solutes, nutrients and oxygen, especially in hydrogels with the highest porosity.

Several strategies have been developed in TE to modify and tune the physical properties of agarose hydrogels to enhance their biological performance. These include the generation of micropatterns on the surface of these materials to favor cell attachment, alignment and differentiation, the generation of a highly porous internal structure by freeze-drying or gas foaming and the development of hydrogels containing gradients of agarose concentration within a single construct, enabling the recreation of heterogeneous environments [33].

Among the most important physical characteristics of agarose biomaterials, their biomechanical and optical properties are particularly relevant, as they can strongly influence the biological response and in vivo suitability of agarose hydrogels for TE applications [16]:

- Biomechanical properties. The biomechanical behavior of agarose hydrogels represents a key physical characteristic, especially when these biomaterials are used in biomedicine. In general, biomaterials applied to biomedicine are exposed to forces and mechanical loads associated with living organisms, such as tissue deformation, traction and compression forces. In these conditions, biomaterials must exhibit suitable stiffness, elasticity, toughness, and structural stability to withstand such stresses without losing their structural integrity [34]. Therefore, the ability of agarose hydrogels to maintain their integrity and functionality under physiological loading conditions is a critical factor determining their suitability for biomedical applications. Among the main biomechanical properties, the ideal biomaterial should have appropriate stiffness, to support incoming forces without excessive deformation, and appropriate elasticity, to recover its original shape after partial deformation, biomimetically reproducing the values displayed by native tissues [35].
   In this regard, agarose biomaterials should have adequate Young’s modulus, which is related to the stiffness of the material under tensile or compressive loading and reflects its ability to resist elastic deformation. Matching the Young’s modulus of the target tissue is often essential to ensure proper biomechanical integration and functionality [36]. In addition, these biomaterials should be able to mimic the viscoelastic behavior of native tissues, which are commonly characterized by the storage modulus (G′), defined as the ability of the biomaterial to elastically recover its original shape after deformation, and the loss modulus (G″), defined as the energy dissipated as heat during loading. Together, these parameters provide valuable information on the balance between elastic and viscous behavior and are critical for predicting the performance of agarose hydrogels under physiological loading conditions [37].
   Different types of agaroses may display different biomechanical properties, reflecting their specific physicochemical profiles (Table 1). In fact, the analysis of five agarose types revealed significant differences among agaroses [16]. Specifically, two standard agaroses (D1LE and D2LE) with a hysteresis range of 36.6–88.3 °C and 40.6–87.8 °C, respectively, generated hydrogels with high Young’s modulus, stress at fracture and break load, indicating high stiffness. In addition, a high-resolution agarose (MS8), generated by reducing the pore size of standard agarose, had a hysteresis of 34–78.5 °C and showed intermediate stiffness, with high strain at fracture. In turn, a high-strength agarose (D5) with hysteresis 36.2–88.1 °C generated very stiff hydrogels, with high Young’s modulus, stress at fracture and break load. Finally, a low-melting point agarose (LM) with hysteresis 26.1–64.9 °C generated low-stiffness hydrogels, with low Young’s modulus, stress at fracture and break load. Together, these results demonstrate that agarose type substantially influences hydrogel stiffness and elasticity, enabling fine-tuning of biomechanical performance for specific TE applications. However, the parameter that most directly influenced the biomechanical properties of agarose hydrogels was concentration, with the stiffer biomaterials corresponding to the highest concentrations of agarose [16].
   Interestingly, other factors may significantly influence the biomechanical properties of agarose hydrogels, including the temperature and velocity of gelation, the presence of cells or the previous thermal story of agarose solutions that may induce hydrolysis, oxidation and intermolecular condensation of agarose [38,39,40]. Therefore, the structural and mechanical properties of agarose hydrogels can be tuned by controlling the jelling conditions, particularly, the temperature and gelation time [40].
   It is well known that the physical properties and three-dimensional structure of biomaterials used in tissue engineering can significantly influence cell physiology and cell proliferation [41]. In particular, parameters such as biomaterial stiffness play a key role in regulating cell–matrix interactions and cell behavior [42]. Therefore, controlling the biomechanical properties of agarose biomaterials may allow researchers to modulate the cellular microenvironment, therefore influencing stem cell fate and differentiation. In addition, tuning the properties of agarose hydrogels and functionalization with bioactive molecules could allow the generation of agarose biomaterials with enhanced biological properties, including improved cell attachment, cell differentiation and more physiological cell–biomaterial interactions [43,44]. In fact, it has been demonstrated that the biomechanical properties of biomaterials can regulate mechanotransduction pathways, which in turn influence signaling processes involved in cell differentiation, supporting the design of biomaterials with definite properties able to promote tissue regeneration [45,46].
   As compared with other biomaterials commonly used in TE, agarose hydrogels typically show higher elastic moduli, making them promising candidates for the development of tissues and organs with stringent elastic requirements. In this regard, Pollot et al. (2018) found that the elastic modulus of hydrogels made with collagen, fibrin, collagen-chitosan and agarose was 3.7 ± 1.2 MPa, 3.3 ± 0.5 MPa, 2.7 ± 1.2 MPa and 87.3 ± 32.6 MPa, respectively. However, agarose was also the most brittle biomaterial [47].
- Optical properties. Analysis of the optical behavior of hydrogels is very important for biomaterials used in specific applications requiring definite transparency levels, such as cornea TE [48]. In general, all biomaterials should have stable optical properties, since these properties are directly related to the structural stability of biomaterials and native tissues [49], and biomaterials used in TE should meet key optical properties in order to ensure adequate function and biological performance. In addition, some tissues and organs, such as the human cornea, should be transparent, while other organs, such as the human skin, should display specific colors [50].
   As highly transparent biomaterials, agarose hydrogels generally exhibit high light transmittance, a parameter defined as the fraction of incident light that can successfully go through a biomaterial without being absorbed or reflected, due to the high homogeneity and structural uniformity of their polymer network [49]. Agarose hydrogels show low reflectance, meaning that only a small percentage of the incident light returns from their surface, low scattering, corresponding to the amount of light that suffers redirection inside the biomaterial due to internal heterogeneities, and low absorbance, defined as the fraction of light energy taken up by the material and converted into other forms of energy, such as heat or chemical excitation within the biomaterial. Together, these properties make agarose an ideal optical medium for TE applications requiring clear visualization and controlled light propagation.
   Few studies have investigated the specific optical properties of agarose hydrogels. In this regard, Seow et al. reported that hydrogels made with standard agarose combined with fish-derived gelatin had a transmittance coefficient of 96% [51]. Moreover, our research group found that fibrin-agarose hydrogels generated with a final concentration of 0.1% low-melting point agarose exhibited high transmittance of visible light, with more than 70% of the incoming light transmitted through the hydrogel. These biomaterials showed very low coefficients of reflectance (<0.1%), absorption (approximately, 1.0 mm^−1^) and scattering (approximately, 2.2 mm^−1^). Regarding ultraviolet (UV) radiation, low-melting point agarose hydrogels combined with fibrin and corneal cells were shown to effectively absorb most harmful UV light in a model of human bioartificial corneal stroma generated by TE [52]. When agarose was used at a final concentration of 0.1%, the absorption coefficient was approximately at 0.7 mm^−1^ for UV-C, 1.8 mm^−1^ for UV-B and 1.7 mm^−1^ for UV-A, with a significant reduction in these coefficients when agarose was used at a final concentration of 0.2% (0.1 mm^−1^ for UVC, 0.9 mm^−1^ for UV-B and 0.8 mm^−1^ for UV-A) [52]. In fact, the excellent optical properties of agarose biomaterials led some researchers to propose the use of agarose-based optical fibers able to efficiently transmit visible light in vivo [53].
   When the optical properties of agarose biomaterials were compared with other natural biomaterials used in TE, agarose has been suggested to exhibit very high optical transparency. This property is likely due to the homogeneous structure of agarose polymer networks, which minimizes light scattering and absorption, and promotes light transmittance [53]. In contrast, fibrillar biomaterials, such as collagen and fibrin, tend to exhibit lower transmittance and high scattering, since these parameters are directly influenced by the three-dimensional organization of the biomaterial fibrillar network [54].

## 6. Biocompatibility of Agarose Biomaterials

Biocompatibility [55] refers to the ability of a material to perform with an appropriate host response in a specific situation, although this concept has been expanded to include long-term co-existence and interactions of biomaterials and tissues in a sustainable manner. As a natural biomaterial, agarose hydrogels are considered to be highly biocompatible, and their application in living organisms is not associated with any adverse or toxic effects.

Biocompatibility of agarose biomaterials has been evaluated both ex vivo, with living cells kept in culture conditions, and in vivo, after implantation in a living organism. In both cases, agarose was demonstrated to be biocompatible.

**Figure 3:**
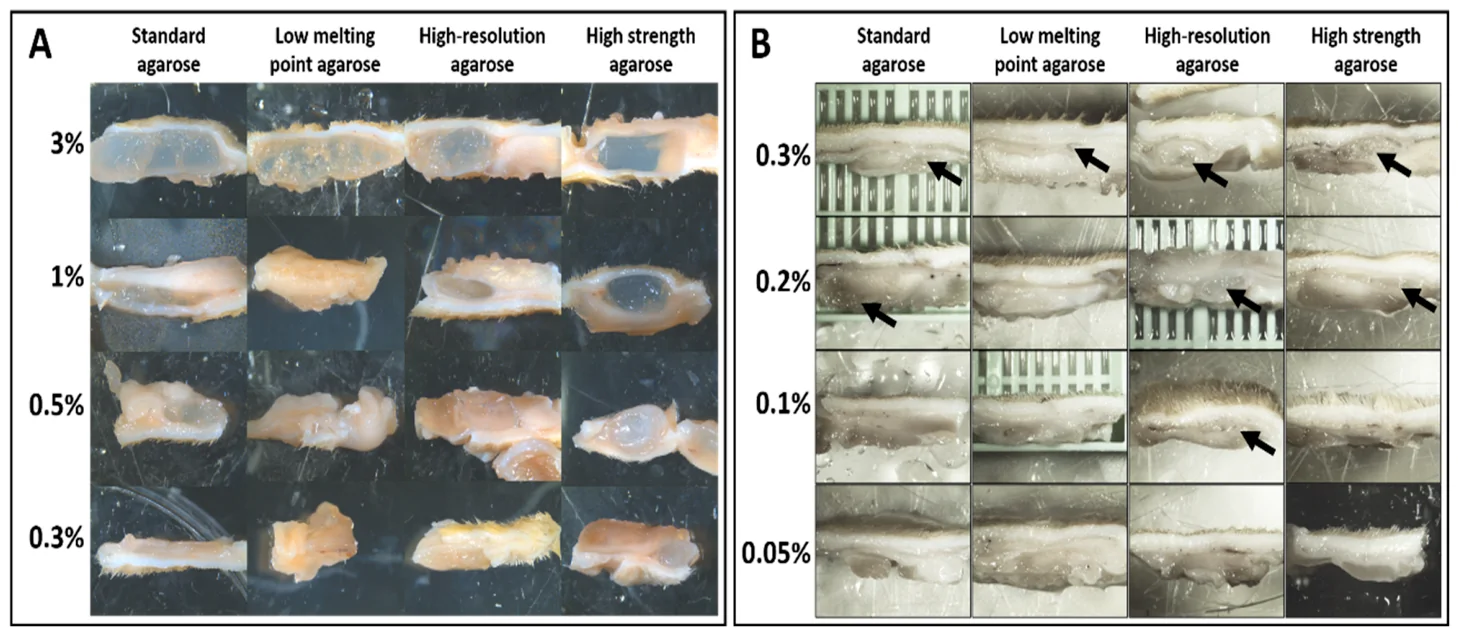
Results of the subcutaneous implants of different types of agarose hydrogels in laboratory animals, showing the macroscopic appearance of each hydrogel. (**A**): Implants of standard agarose, low-melting point agarose, high-resolution agarose and high strength agarose hydrogels generated at different concentrations (3%, 1%, 0.5% and 0.3%), after 3 months of in vivo follow-up. (**B**): Implants of fibrin-agarose hydrogels generated with human fibrin combined with standard agarose, low-melting point agarose, high-resolution agarose and high strength agarose containing different concentrations of agarose (0.3%, 0.2%, 0.1% and 0.05%), after 1 month of in vivo follow-up. Specific areas of agarose identifiable in the animal tissues are labeled with arrows. Adapted from Irastorza-Lorenzo et al. (2021), Int J Mol Sci 22, 1923 [16], and Ortiz-Arrabal et al. (2023), Mar Drugs 21, 187 [57], licensed under CC BY 4.0.

- Ex vivo biocompatibility. It has been demonstrated that agarose hydrogels are very well tolerated by cultured cells and show high cytocompatibility. In fact, ex vivo evaluation of different types of agarose hydrogels using different analysis methods demonstrated high biocompatibility, with no detectable adverse effects on cultured cells. Specifically, agarose hydrogels cultured in indirect contact with human cells were not able to impair cell viability, metabolic activity or proliferation, suggesting that these biomaterials did not release cytotoxic components into the surrounding medium. Additionally, cells cultured within the different types of agaroses remained viable and metabolically active, although the highest concentrations of agarose limited cell proliferation, most likely due to the increased stiffness and reduced porosity of the denser hydrogels [16]. Overall, these results confirm that agarose is a highly biocompatible scaffold for cells cultured ex vivo. The fact that agarose is biologically inert and lacks intrinsic cell adhesion motifs reduces inflammatory and toxic signaling, but limits cell adhesion and spreading.
- In vivo biocompatibility. Adequate in vivo behavior is a requirement for biomaterials used in TE, and a thorough evaluation in laboratory animals is essential to determine the potential usefulness of agarose biomaterials. In this sense, the subcutaneous implants of different agarose types on laboratory rats demonstrated that all the evaluated agaroses were safe, with no local or systemic adverse effects identified in vivo [16]. Agarose did not elicit a detectable inflammatory reaction, and its degradation was mainly mediated by pro-regenerative M2 macrophages. Agarose biomaterials are often considered as minimally degradable in vivo, because human tissues lack agarases and other enzymes able to degrade agarose [56]. Instead, agarose is degraded in vivo by means of oxidation, hydrolysis and other slow degradation pathways requiring extended time periods, which explains the stability of the material once grafted in vivo, as well as its biocompatibility. Interestingly, composite fibrin-agarose biomaterials also showed excellent in vivo biocompatibility, although biodegradation was more intense than in agarose biomaterials alone [57]. In both cases, the most relevant factor influencing reabsorption of the grafted biomaterial was concentration, with the highest concentrations of agarose resulting in lower levels of biodegradation after 1 to 3 months of follow-up (Figure 3). Overall, these findings suggest that agarose biomaterials show higher in vivo stability than other natural biomaterials, such as fibrin or collagen, and may be more resistant to biodegradation once grafted in vivo. In contrast, agarose biomaterials do not contain cell adhesion and cell differentiation motifs that are present in fibrillar extracellular matrix proteins, such as fibrin and collagen, resulting in a more inert environment as compared to fibrin and collagen hydrogels [10].

## 7. Biofabrication Methods of Agarose Hydrogels in TE

In TE, agarose biomaterials have been widely used, either as single materials or combined with other biomaterials using different biofabrication strategies. Due to their thermoresponsive behavior, biomechanical tunability and biocompatibility, agarose biomaterials can be processed using multiple fabrication approaches that enable precise control over three-dimensional architecture, cell distribution, and matrix organization.

This chapter describes the main TE biofabrication strategies involving agarose-derived biomaterials, including processing principles and manufacturing methods, whereas specific tissue and organ applications are described in Section 8.

- Generation of microtissues and organoids. Organoids are three-dimensional structures generated in the laboratory that recapitulate the minimal structure and functions of a native organ, whereas microtissues are formed by a single cell type and recapitulate the structure of a native tissue, but lack the multi-lineage complexity of organoids [58]. Although the biofabrication processes described for these structures are highly variable, one of the most commonly used protocols is culturing cells on micromolds made with highly concentrated agarose. As an inert biomaterial, agarose favors cell assembly and the formation of three-dimensional cell aggregates within the microwells generated in agarose micromolds [59]. This simple method allowed for the generation of different types of aggregates, such as chondrocyte [60] and stem cell microtissues [61].
- Fabrication of agarose beads. Agarose biomaterials can be used to generate beads, microbeads and nanobeads. These scaffolds may have numerous applications, such as carriers and nanoformulated drugs and other compounds [62], magnetic particles able to absorb specific products [63] or cell encapsulation [64], among others.
- 3D Bioprinting. As a biomaterial whose physical state mainly depends on temperature, agarose biomaterials can be fabricated by 3D bioprinting. In fact, this technology allowed for the generation of diverse biological substitutes [65], and its applications are expected to be significantly expanded in the future. However, specific modifications are frequently done in the chemical structure of agarose to favor 3D printing, such as metacrylation, combination with other types of biomaterials or crosslinking [66,67].
- Generation of bioartificial tissues by TE using biomaterials generated de novo. One of the most widely used strategies to fabricate bioartificial tissues and organs is de novo assembly biofabrication, which combines cells, biomaterials and bioactive factors or specific biofabrication methods [68]. In this approach, cells are incorporated into liquid agarose biomaterials, which are then aliquoted and allowed to jellify, and epithelial cells can be later cultured on the surface of this biomaterial. This methodology has supported the generation of certain types of tissues and organs. However, the low biological interaction of agarose hydrogels with cells motivated several researchers to generate composite biomaterials in which agarose is combined with other biologically active biomaterials or bioactive components. These hybrid systems enhance cell adhesion, proliferation, and differentiation, thereby improving the biological performance and translational potential of agarose-based constructs. The main composite materials containing agarose are summarized in Table 2.

## 8. Applications of Agarose Biomaterials in TE

Due to their excellent biocompatibility, structural stability and physical properties, agarose-based biomaterials have been successfully used for the generation of a wide range of bioengineered tissues and organs (Table 2). By applying the biofabrication methods described in the previous chapter, agarose-based biomaterials have been applied to the fabrication of biomimetic substitutes capable of reproducing the structure and function of native tissues and organs. The resulting tissues and organ models have demonstrated high biocompatibility, functional relevance, and promising translational potential, establishing agarose as a key biomaterial in the development of clinically meaningful TE applications. In addition to the generation of tissue and organ substitutes, agarose biomaterials have been employed in several other applications, including serving as a supporting niche for the generation of microtissues and organoids, facilitating three-dimensional cell assembly, and functioning as hemostatic agents.

**Figure 4:**
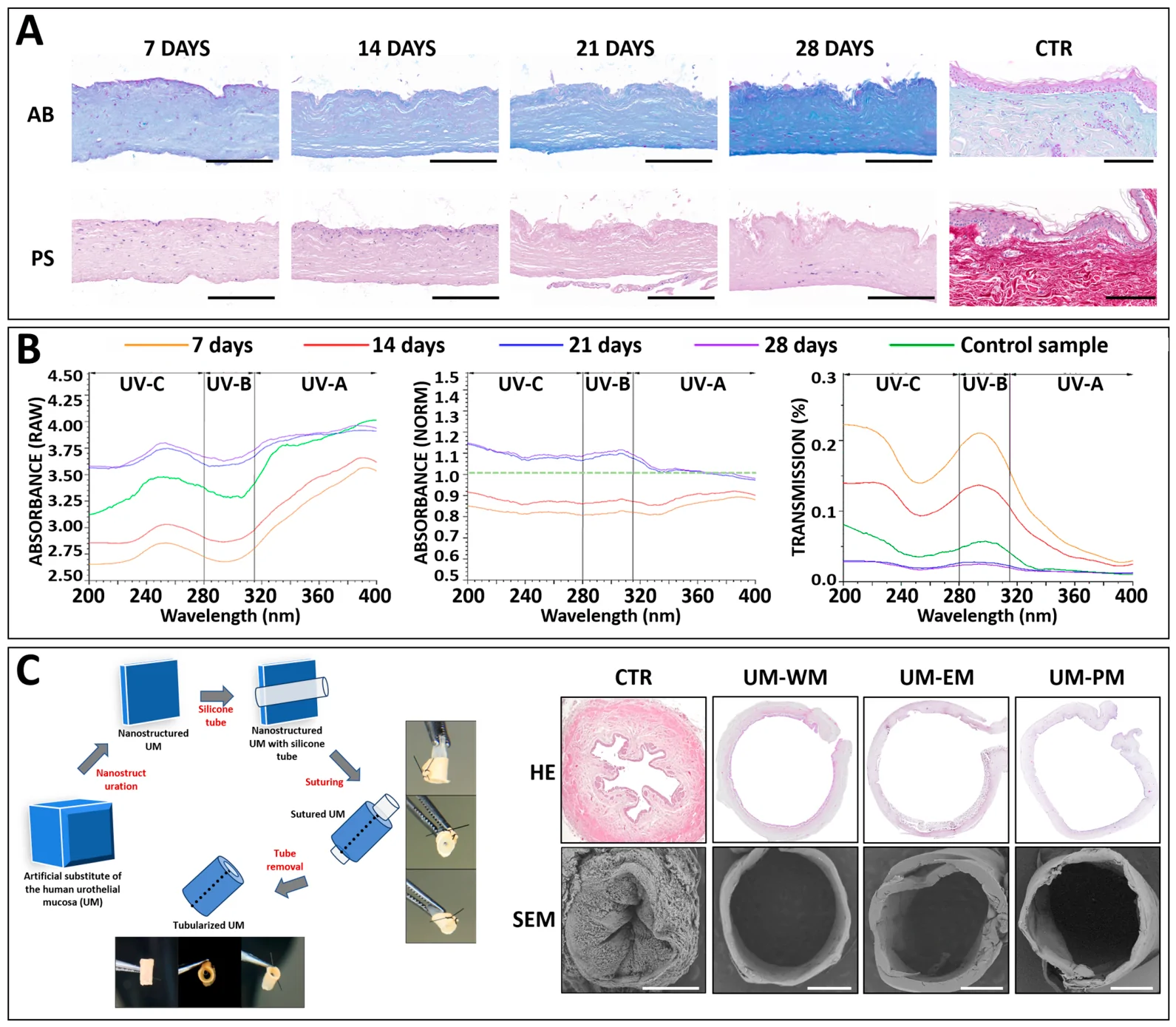
Representative applications of fibrin-agarose hydrogels in the generation of bioartificial tissues by TE. (**A**): Histological images of the UGRSKIN human artificial skin generated with low-melting point agarose at a concentration of 0.1% stained with alcian blue (AB) and picrosirius red (PS), after 7, 14, 21 and 28 days of development in culture, and human control native skin (CTR). Scale bars: 200 µm. (**B**): Optical characterization of UGRSKIN after 7, 14, 21 and 28 days of development in culture, and human control native skin, showing raw and normalized absorbance and transmission parameters. (**C**): Generation of tubularized substitutes of the human urothelial mucosa (UM), showing the biofabrication procedure and low-magnification histological images of control human UM (CTR) and tubularized substitutes of the human UM cultured with three different media conditions (WM, EM and PM), stained with hematoxylin-eosin (HE) and analyzed with scanning electron microscopy (SEM). Scale bars: 1 mm. Adapted from Ruiz-López et al., 2022 [109], licensed under CC BY 4.0, and Garzón et al., 2021 [102], licensed under CC BY 4.0.

The main applications of agarose-based biomaterials in TE include:

- Cartilage. Agarose biomaterials have been widely used in cartilage TE, due to the physicochemical properties of agarose hydrogels and their ability to mimic the intrinsic characteristics of human cartilage. In this regard, agarose scaffolds can be combined with chondrocytes to generate a substitute of the human cartilage or can be injected at damaged joints to induce cartilage regeneration [9]. In addition, cartilage substitutes have been generated using 3D-printing strategies [89], whereas substitutes of the intervertebral disk have been reported by incorporating chondrocytes into agarose scaffolds [90].
- Bone. In this case, agarose biomaterials have been combined with hydroxyapatite and subjected to supercritical gel drying, and combined with bone cells to generate promising substitutes of osteogenic structures [88]. In addition, agarose can be subjected to 3D printing to generate efficient bone substitutes by TE [91].
- Tendon. Agarose have been combined with different biomaterials, such as collagen, to generate nice models of tendons able to partially reproduce the three-dimensional structure of the native tissues [92].
- Adipose tissue. Combined with adipose tissue-derived stem cells or with differentiated adipocytes, agarose hydrogels allow for the efficient generation of adipose tissue substitutes with adequate biomechanical and functional properties [93].
- Skin. Several models of the human skin have been developed using agarose biomaterials fabricated de novo or using 3D printing [94]. Among others, UGRSKIN (Figure 4) was generated by combining agarose with human fibrin, providing this biomaterial with increased biocompatibility that was subjected to plastic compression nanostructuration, resulting in a highly biomimetic substitute of the human skin that showed promising results in severely burnt patients [84]. These positive results allowed for the authorization of UGRSKIN as the first tissue engineering ATMP approved for clinical use in Spain.
- Cornea. By combining agarose with human fibrin and the three main cells of the cornea, researchers were able to generate the first thee-layered model of the rabbit cornea [12]. In addition, the same biomaterial allowed researchers to generate NANOULCOR, a biomimetic model of the human anterior lamellar cornea that showed promising results in a pioneering clinical trial carried out in patients with corneal blindness (EU CT number: 2023-506856-25-00) [95].
- Sclera. As the external fibrous part of the human eye, the sclera is frequently affected by diverse conditions that require grafting a biological substitute capable of reproducing the functions of the native sclera. One of the tissue-engineered products that showed promising preclinical results is fibrin-agarose, which allowed the efficient regeneration of the rabbit sclera in animal models, demonstrating adequate integration and potential to restore tissue architecture [96].
- Oral and palate mucosa. Nanostructured fibrin-agarose biomaterials were used to generate BIOCLEFT, a bilayered substitute of the human oral and palate mucosa showing promising results in a clinical trial implemented in children with cleft lip and palate (ClinicalTrials.gov Identifier NCT06408337) [97].
- Peripheral nerve. Combined with aniline, researchers generated a biomaterial with electroactive properties potentially useful in neural tissue engineering [98]. In addition, nanostructured fibrin-agarose with adipose stem cells cultured within this biomaterial demonstrated in vivo regenerative potential in animal models [98,99].
- Urological structures. In the field of urology, agarose has been successfully applied to the generation of bioartificial substitutes of the human bladder mucosa [100], urethra [101] and ureter [102] (Figure 4). These structures demonstrated adequate histological structure and allowed differentiation of the cells cultured within the biomaterial. Moreover, agarose gel stands have been employed as supportive platforms for the development of testicular tissue, providing a stable environment facilitating cell organization [103].
- Other applications of agarose biomaterials. Agarose-based hydrogels have also been explored in several complementary applications in the field of TE. Agarose has been used as a carrier biomaterial for the injection of bioactive factors aimed at promoting vascular regeneration [104], and as a support medium for bioprinting applications requiring stable, cell-compatible platforms [105]. Additionally, agarose-gelatin cryogels have been applied to the generation of several types of soft tissues in the laboratory [106], demonstrating suitable porosity and biocompatibility. Fibrin-agarose biomaterials have shown preliminary in vivo efficacy as hemostatic agents [107] and have been used in abdominal wall reconstruction in laboratory animals [108].

## 9. Conclusions

Agarose-based biomaterials have demonstrated potential usefulness in TE. As a thermoreversible, autoclavable biomaterial, agarose hydrogels offer the opportunity to generate tissue substitutes containing embedded cells and other cell types on their surface, allowing for the generation of complex, multilayered tissues and organs. Its molecular architecture, microstructural organization, mass-transport properties, and chemical inertness position this biomaterial as a highly adaptable matrix for the generation of bioartificial tissues requiring controlled mechanics, optical performance, and robust biocompatibility. Compared with other biomaterials, agarose hydrogels exhibit high structural stability and excellent biomechanical and optical properties. However, agarose is intrinsically bioinert and lacks the cell adhesion and signaling molecules found in fibrin, collagen and other fibrillar natural biomaterials. These limitations can be overcome by combining agarose with other biologically active biomaterials, such as fibrin [15], resulting in a composite product able to integrate the favorable physical properties of agarose with the biocompatibility of natural fibrillar biomaterials. These properties make agarose a suitable biomaterial for TE of tissues characterized by a dense extracellular matrix and cells with reduced metabolic activity, such as human cartilage. Agarose is also an ideal component of composite biomaterials requiring additional optical and biomechanical properties, in combination with fibrillar biomaterials, for use in TE of the human skin, cornea, oral and urinary mucosa.

The use of agarose biomaterials is hindered by several challenges, including variable biodegradation profiles, the need to ensure reproducibility and standardization across batches, methods able to scale-up production, and clinical translation. Future research should address these issues to expand the use of agarose biomaterials in TE and regenerative medicine. On the one hand, reproducibility and standardization is an important requirement for ATMP generated in the laboratory, since it allows for alignment with regulatory frameworks and large-scale manufacturing [110]. However, reproducibility is an inherent difficulty when working with natural biomaterials extracted from biological sources whose nature is highly variable and batch-dependent, as is the case with agarose [111]. Furthermore, scaled-up production of agarose-derived biomaterials is challenging, and several studies focused on the generation of agarose beads have shown that large-scale manufacturing is complex and requires strict control of process parameters, and mathematical modeling has been proposed as a promising strategy supporting industrial-scale production [112].

Moreover, one of the most challenging steps in the application of products generated by TE is clinical translation, and only a very small number of bioartificial tissues and organs have successfully reached the clinical setting. This limitation is largely attributable to the highly complex regulatory frame controlling clinical translation of ATMP, which imposes strict requirements on safety, efficacy and quality controls [113]. In addition, novel therapies generated by TE often fail to reach the clinical setting due to combined scientific, industrial, regulatory and economic barriers [114]. In this regard, some agarose biomaterials, especially those combined with fibrin, have already been authorized by a Medicines Agency for clinical use (e.g., the artificial skin product UGRSKIN), and others are currently being evaluated in ATMP clinical trials (e.g., NANOULCOR EU CT number: 2023-506856-25-00 and BIOCLEFT ClinicalTrials.gov Identifier NCT06408337). The preliminary clinical results obtained with these products suggest that fibrin-agarose biomaterials can be efficiently used to generate bioengineered substitutes of the human skin, cornea and palate mucosa, allowing for the successful treatment of patients with severe burns, corneal blindness and cleft palate [84,95,97].

These achievements underscore the translational potential of agarose biomaterials and justify further exploration of their applicability to additional therapeutic contexts. Future studies should determine the potential clinical usefulness of these biomaterials for other therapeutic applications. The unique properties of agarose position this biomaterial as a promising platform in next-generation tissue engineering.

## Figures and Tables

**Table 1:** Biomechanical characteristics of five agarose types. The table includes hysteresis ranges, gel strength values, and the main biomechanical parameters (Young’s modulus, stress at fracture, strain at fracture and break load) measured across concentrations from 0.3% to 3%, highlighting the distinct stiffness and elasticity profiles that characterize each agarose type. The values on the right correspond to the average values obtained experimentally for the main biomechanical parameters of each type of agarose, calculated in a single study published by Irastorza-Lorenzo et al. (2021) [16].

Agarose Name	Agarose Type	Key Physicochemical Data	Biomechanical Profile	Young Modulus (Mpa)	Stress at Fracture (MPa)	Strain at Fracture (mm/mm)	Break Load (N)
D1LE	Standard agarose/High-strength agarose	Hysteresis 36.6–88.3 °C; gel strength 2990 g/cm^2^ (1.5%)	High Young’s modulus, high stress at fracture, high break load; medium strain at fracture	0.12 ± 0.16	0.01 ± 0.02	0.31 ± 0.14	27.57 ± 38.45
D2LE	Standard agarose/High-strength agarose	Hysteresis 40.6–87.8 °C; gel strength 2310 g/cm^2^ (1.5%)	High stiffness; rapid gelation; medium strain at fracture	0.13 ± 0.17	0.01 ± 0.02	0.24 ± 0.07	28.35 ± 38.28
LM	Low-melting point agarose	Hysteresis 26.1–64.9 °C; gel strength 1100 g/cm^2^ (1%)	Softest hydrogel; fails to gel at 0.3%; lowest stiffness	0.10 ± 0.10	0.01 ± 0.01	0.21 ± 0.05	14.87 ± 13.41
MS8	High-resolution agarose	Hysteresis 34–78.5 °C; gel strength 3590 g/cm^2^ (1.5%)	Intermediate stiffness; highest strain at fracture (most elastic)	0.08 ± 0.10	0.01 ± 0.01	0.27 ± 0.04	21.25 ± 29.03
D5	High-strength agarose	Hysteresis 36.2–88.1 °C; gel strength 4120 g/cm^2^ (1.5%)	High Young’s modulus, high stress at fracture, high break load; high deformability	0.12 ± 0.15	0.02 ± 0.02	0.32 ± 0.08	32.22 ± 36.23

**Table 2:** Major agarose-based composite biomaterials, highlighting the key functional contribution of each added component, some reported applications, and representative references. The summary illustrates how combining agarose with bioactive or mechanically tunable materials enhances its performance in TE and regenerative medicine.

Composite	Description and Functional Contribution	Applications	References
Agarose–Collagen	Collagen provides cell-adhesive motifs (RGD, GFOGER), improving cell attachment and migration; agarose contributes mechanical stability and optical clarity.	Corneal, neural, skin, cartilage tissue engineering.	López-Marcial et al., 2022 [69]; Wang et al., 2024 [70]
Agarose–Gelatin	Gelatin adds bioactivity, MMP-sensitive degradability, and cell-binding sites; agarose maintains shape fidelity and reduces contraction.	3D bioprinting, soft tissue scaffolds, cell encapsulation.	Sakai et al., 2007 [71]; Dravid et al., 2022 [72]
Agarose–Alginate	Alginate enables ionic crosslinking, tunable stiffness, and rapid gelation; agarose improves mechanical robustness and transparency.	Cell encapsulation, microgels, drug delivery.	Xiang Ping et al., 2023 [73]; Atoufi et al., 2017 [74]
Agarose–Chitosan	Chitosan provides antimicrobial activity, positive charge, and bioactive signaling; agarose stabilizes the matrix and modulates swelling.	Wound healing, skin, cartilage repair.	Sang et al., 2023 [75]; Saeedi et al., 2020 [76]
Agarose–Polyacrylamide	Highly tunable mechanical properties for mechanobiology; agarose maintains optical clarity for imaging.	Mechanotransduction studies, soft implants, cell biomechanics.	Taylor & Penhallow, 1986 [77]; Guo et al., 2018 [78]
Agarose–PEG	PEG increases biocompatibility, reduces protein adsorption, and allows chemical functionalization; agarose provides structural support.	Controlled drug delivery, cell encapsulation.	Sartip et al., 2025 [79]
Agarose–Hyaluronic Acid	HA enhances hydration, cell signaling, and matrix elasticity; agarose stabilizes the hydrogel and reduces degradation rate.	Cartilage, skin, wound regeneration, ocular TE.	Guo et al., 2025 [80]; Chu et al., 2020 [81]
Agarose–Nanoparticle Composites	Incorporates silica, graphene oxide, gold nanoparticles, or nanocellulose to improve mechanical strength, conductivity, or sensing capabilities.	Bioelectronics, reinforced scaffolds, biosensing, 3D bioprinting.	Kumar et al., 2018 [82]; Nadernezhad et al., 2019 [83]
Agarose–Fibrin	Fibrin provides strong bioactivity, cell adhesion, and pro-regenerative signaling; agarose improves mechanical integrity and reduces contraction.	Skin, cornea, palate, peripheral nerve, oral mucosa.	Alaminos et al., 2006 [12]; Martín-Piedra et al., 2023 [84]
Agarose-polycaprolactone	Biodegradable, cytocompatible, pH-responsive. Injectable, tunable mechanics.	Drug delivery, tissue engineering.	Chandel et al., 2016 [85]; Ghaedamini et al., 2023 [86]
Agarose-hydroxyapatite	Biocompatibility, adhesion, coagulation. Osteoconductive and hemostatic	Bone regeneration.	Tabata et al., 2005 [87]; Zanotti et al., 2024 [88]

## Data Availability

No new data were created or analyzed in this study. Data sharing is not applicable to this article.

## Abbreviations

The following abbreviations are used in this manuscript:

ATMP	Advanced Therapy Medicinal Product
BIOCLEFT	Fibrin-Agarose Artificial Palate Mucosa
ECM	Extracellular Matrix
G′	Storage Modulus
G″	Loss Modulus
NANOULCOR	Fibrin-Agarose Artificial Cornea
PBS	Phosphate-Buffered Saline
RGD	Arginine–Glycine–Aspartic Acid peptide
TAE	Tris–Acetate–EDTA
TBE	Tris–Borate–EDTA
TE	Tissue Engineering
UGRSKIN	Fibrin-Agarose Artificial Skin
UV	Ultraviolet light
